# Surfactant-Free Synthesis of Nb_2_O_5_ Nanoparticles Anchored Graphene Nanocomposites with Enhanced Electrochemical Performance for Supercapacitor Electrodes

**DOI:** 10.3390/nano10010160

**Published:** 2020-01-17

**Authors:** P. Nagaraju, R. Vasudevan, A. Alsalme, A. Alghamdi, M. Arivanandhan, R. Jayavel

**Affiliations:** 1 Centre for Nanoscience and Technology, Anna University, Chennai-600025, Tamil Nadu, India; nanonaga85@gmail.com (P.N.); rvasu85@gmail.com (R.V.); arivucz@gmail.com (M.A.); 2 Department of Physics, School of Arts and Science, AV campus, Vinayaka Mission’s Research Foundation, Chennai-600105, Tamil Nadu, India; 3 Department of Chemistry, College of Science, King Saud University, P.O. Box 2455, Riyadh Province-11451, Riyadh, Saudi Arabia; aalsalme@ksu.edu.sa (A.A.); aalghamdia@ksu.edu.sa (A.A.)

**Keywords:** nanocomposites, electrode material, supercapacitors, charge transport, cyclic stability

## Abstract

Nb_2_O_5_/graphene nanocomposites without any surfactant are synthesized by an in situ microwave irradiation technique. Structural and morphological studies revealed that the prepared composites were composed of Nb_2_O_5_ nanoparticles intercalated into the graphene sheet. The thermal stability of graphene oxide, Nb_2_O_5_, and Nb_2_O_5_/graphene nanocomposite was studied by the TGA. The electrochemical properties are assessed by cyclic voltammetry, chronopotentiometry and electrochemical impedance spectroscopy analyses. The specific capacitance of Nb_2_O_5_/graphene nanocomposites is greater (633 Fg^−1^) than pure Nb_2_O_5_ nanoparticles (221 Fg^−1^) and graphene (290 Fg^−1^) at a current density of 1 Ag^−1^. The long-term cyclic measurement confirms higher cyclic stability of the nanocomposite with capacitance retention of 99.3% after 5000 cycles without performance degradation. The composites exhibit higher electrochemical conductivity and allow effective ions and charge transport over the entire electrode surface with aqueous electrolyte. The electrochemical study suggests that Nb_2_O_5_/graphene nanocomposites have the potential to be an effective electrode for superior performance supercapacitor applications.

## 1. Introduction

In recent years, the depletion of natural energy sources due to the industrialization of technological advancements has been alarming. Among the most important requirements is the judicious use of available energy, and an efficient mechanism for energy storage [1]. Renewable energy sources are great replacements for conventional fossil fuels [2]. The predicted future energy crisis demands efficient energy conversion and storage devices [3,4]. Electrochemical capacitors, known as supercapacitors, possess the high power output of conventional dielectric capacitors and the high energy storage of batteries. The current challenge is to sustainably achieve higher power density and instant charge-discharge [5,6]. In general, electrochemical capacitors can be divided into two categories, depending on the storage process. The first type of Supercapacitor is termed the pseudocapacitor, with charge storage by Faradic reactions [7]. Materials under this category include conducting polymers and metal oxides. The second type is the electric double-layer capacitor (EDLC), which involves an electrostatic process for storage. These types of materials include the carbon family, including activated carbon, carbon aerogels, carbon nanotubes, and graphene, which exhibit high surface area. These electrode materials play a vital role in the performance of supercapacitors, based on their electrochemical performance [8,9,10]. Hence, lucid design of efficient materials is a prerequisite for high-performance supercapacitors.

Several materials have been studied as electrodes for supercapacitors in order to improve the energy and power density requirements. Among them, the transition metal oxide Nb_2_O_5_ has been explored recently due to its higher valance state and excellent structural stability with pseudocapacitance behavior. It is widely used in many potential applications, such as solar cells [11], electrochromic materials [12], photocatalysis of water to produce hydrogen [13], gas sensing [14], and especially photo-degradation of harmful organic contaminants in water [15] due to outstanding advantages of low toxicity, thermodynamic stability, and relatively high photocatalytic activity. Nb_2_O_5_ has many forms, such as a-Nb_2_O_5_ (amorphous), TT-Nb_2_O_5_ (pseudohexagonal), T-Nb_2_O_5_ (orthorhombic) and M- Nb_2_O_5_ (monoclinic), which can be obtained through controlled thermal treatment [16]. Structure-dependent electrochemical performance has been investigated on Nb_2_O_5_ and it has been observed that orthorhombic Nb_2_O_5_ (T-Nb_2_O_5_) has comparatively better electrochemical performance than the monoclinic phase [17]. Amorphous and pseudo-hexagonal Nb_2_O_5_ exhibits lower specific capacitance values. 

However, the poor electronic conductivity of Nb_2_O_5_ nanoparticles limits their electrochemical utilization [18]. Additionally, the fabrication of nanoparticles involves heavy agglomeration due to van der Wall’s force that limits the applicability due structural instability. Graphene (G) offers large 2D space with a large surface area for the decoration of nanoparticles for fast ion transport. The 2D structure of G provides suitable platform to accommodate electrochemically active materials. In addition, G also offers opportunities to develop nanocomposites with wide range of materials for diverse applications [19,20].

To date, several methods have been adopted to synthesize Nb_2_O_5_ nanocomposites. Wang et al. reported the Nb_2_O_5_/graphene synthesized by hydrothermal method [21]. T-Nb_2_O_5_/graphene was prepared through a facile hydrothermal method by Kong et al. [22]. Murugan et al. reported a hydrothermal method [23]. However, these methods have been limited by lower capacitance with lower stability of the Nb_2_O_5_/graphene nanocomposite; other disadvantages include the long processing time, exorbitant cost, and higher processing temperature, which would hinder large-scale production. The microwave irradiation method can be used as an alternative heat source for other synthesis methods, leading to fast heating, achieving the desired temperature in a short duration and increasing the reaction kinetics compared with the conventional methods. The supercapacitor properties have generally been studied using organic electrolytes, which are expensive and hazardous. In this study, aqueous electrolyte was used because it is less expensive, while being environmentally friendly and easy to use, and possessing greater ionic conductivity than organic electrolytes, which are required to improve rate capability and high power density. 

In this report, a new approach is demonstrated for synthesizing high-quality Nb_2_O_5_ nanoparticles combined with graphene without any surfactant via a one-step in situ microwave irradiation method. This process is inexpensive, straightforward, and can be readily adopted for the production of larger quantities of nanoparticles. This ultrafast, eco-friendly microwave irradiation method is used to prepare graphene and demonstrated the decoration of G surface with Nb_2_O_5_ nanoparticles. Further heat treatment at 700 °C leads to the reduction of remaining graphene oxide into graphene, while the amorphous Nb_2_O_5_ nanoparticles are recrystallized into T-Nb_2_O_5_ nanoparticles. Furthermore, the microwave irradiation method improves the physico-chemical properties of the T-Nb_2_O_5_/graphene nanocomposite. The synergistic effect of T-Nb_2_O_5_ and G composite exhibits promising properties with higher capacitance and very good sustainability in aqueous electrolyte.

## 2. Experimental Section

### 2.1. Material Synthesis 

High purity natural graphite powder (Alfa easer 99.999%), KMnO4 (SRL Extra pure AR), NaNO_3_ (Merck), H_2_SO_4_ (Merck), H_2_O_2_ (Merck), HCl (Merck), and ammonium niboate (v) oxalate hydrate (C_4_H_4_NbO_9_.XH_2_O) (Aldrich), ammonium hydroxide (NH_4_OH) (Merck) were used for the synthesis of electro-active materials.

Preparation of graphene oxide (GO) by the modified Hummer’s method has already been described in an earlier report [24]. The microwave method was employed to prepare Nb_2_O_5_ nanoparticle-decorated G nanocomposite. In a typical reaction, 80 mg of GO was mixed in 100 mL DI water and sonicated for 2 h. After the sonication, the obtained GO solution was mixed with 0.02 M of ammonium niobate (V) oxalate hydrate by constantly stirring for 60 min. Subsequently, ammonium hydroxide was added to the solution at a pH of 12 with continuous stirring for 30 min to get the brownish solution. This solution was loaded in a microwave oven operated at 850 W for 10 min. followed by natural cooling to RT. Subsequently, 3 mL of hydrazine hydrate was added into the solution and stirred for 1 h followed by heating for 3 min. The final product of greyish black precipitate was filtered, washed successively and dried at 60 °C overnight in hot air oven to improve the crystallinity. The prepared Nb_2_O_5_/G was calcined at 600 °C for 4 h in N_2_ atmosphere. The same process was repeated without GO to synthesize pure Nb_2_O_5_ nanoparticles.

### 2.2. Characterization 

The structural properties of Nb_2_O_5_/G nanocomposite were studied by Rigaku Miniflex (Rigaku Miniflex, Japan) X-ray diffractometer with CuKα radiation in the scan range of 5–80°. The morphology study of the nanocomposite was accomplished by scanning electron microscope (TESCAN VEGA3, Czech Republic) and Transmission electron microscope (FEI Technai, Hitachi, Germany). The presence of functional groups was confirmed by FTIR (Bruker optics systems, Germany) spectral analysis performed by KBr pellet method. Thermal stability (SII-TG/DTA A6300, Japan) of the nanocomposite was studied in N_2_ atmosphere at 20 °C/min in the temperature range RT to 1000 °C. The structural properties of composite were studied by Raman spectral analysis (Lab RAM HR micro Raman system, France). The BET analysis was performed using N_2_ adsorption-desorption isotherms at 77 K using the (Quanta Chrome Instruments (version 6.0), Florida). The binding energy states of the composite were studied by X-ray photoelectron spectroscopy analysis using a (Shimadzu ESCA 3400, India) spectrometer. 

### 2.3. Fabrication of Working Electrode 

To prepare the working electrode, Nb_2_O_5_/G electro-active material was mixed with ethanol and a few drops of nafion paste in the weight ratio of 8:1:1. The mixed solution was sonicated for a few min to achieve the homogeneity. The prepared paste was coated on the glassy carbon electrode. The prepared working electrode was dried at 80 °C for 2 min. The electrolyte was prepared by mixing 1 M H_2_SO_4_ with distilled water and stirring for 30 min. The cyclic voltammograms (CV) and chronopotentiometry (CP) were recorded at the potential range of 0.45–1.0 V at different scan rates of 5 to 100 mVs^−1^ and different current densities. The electrochemical impedance spectroscopy (EIS) was analyzed in the frequency range from 0.01 Hz to 105 Hz using a three-electrode system at room temperature. 

## 3. Results and Discussion

### 3.1. X-ray Diffraction Analysis 

**Figure 1:**
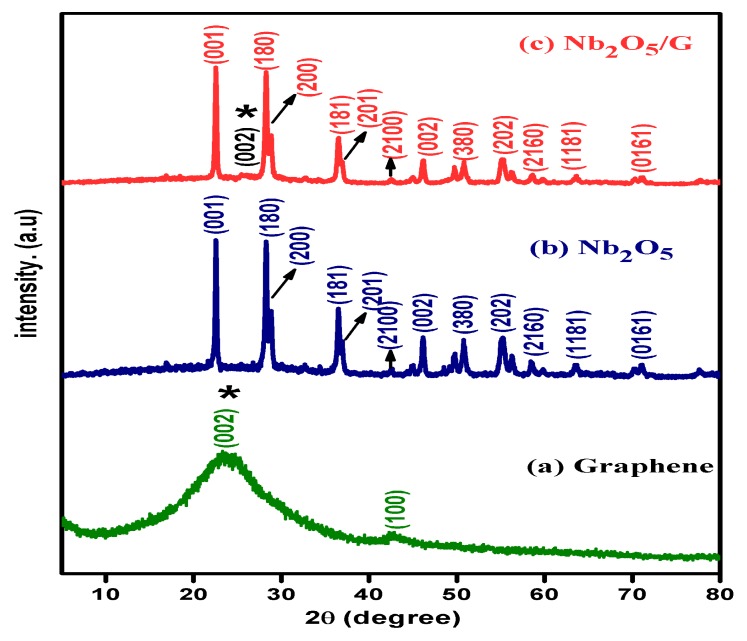
Powder X-ray diffraction patterns of (**a**) graphene, (**b**) Nb_2_O_5_, and (**c**) Nb_2_O_5_/G composite.

Figure 1 shows XRD patterns of G, Nb_2_O_5_, Nb_2_O_5_/G nanocomposites. The diffraction peaks of graphene observed at 2θ = 24.43° and 43.14°, and are very well matched with the data profile of JCPDS No. 75-1621, as shown in Figure 1a. The broad, low-intensity diffraction patterns of (002) and (100) planes were assigned to the hexagonal structure and poor crystalline nature of the sp^2^ bonded carbon. No other diffraction peaks were observed, confirming the removal of oxygen functional groups from the GO by the reduction process. Figure 1b,c reveals the crystalline nature of Nb_2_O_5_, Nb_2_O_5_/G and diffraction peaks are assigned to orthorhombic phase (JCPDS No. 30-0873). The high-intensity peaks belong to Nb_2_O_5_ nanoparticles and Nb_2_O_5_/G composites, confirming the strong crystalline nature of the prepared materials. The (002) diffraction peak of G in Figure 1c also confirmed the formation of Nb_2_O_5_ decorated nanocomposite. 

### 3.2. Scanning Electron Microscopy Investigations

**Figure 2:**
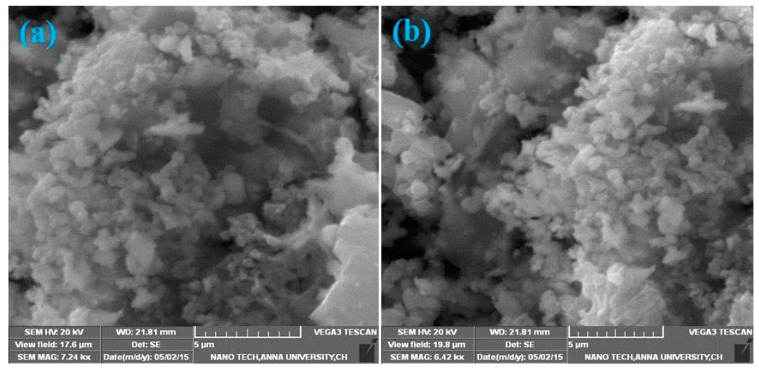
(**a**,**b**) Morphology of Nb_2_O_5_/G nanocomposite annealed at 600 °C.

The SEM images (Figure 2a,b) show Nb_2_O_5_ nanoparticle-incorporated graphene sheets. The coagulation of the nanoparticles was controlled by the polar oxygenated functional groups, which served as preferred sites for Nb_2_O_5_ nanoparticles. The calcination process leads the nanoparticles to become aggregated, forming a porous structure with rough surface [25]. The images show that the G sheets are assembled by swelling radially from the center. This swelling offers a relatively large contact area at the electro-active material–electrolyte interface, providing short and more efficient ion transport. 

### 3.3. TEM Analysis

**Figure 3:**
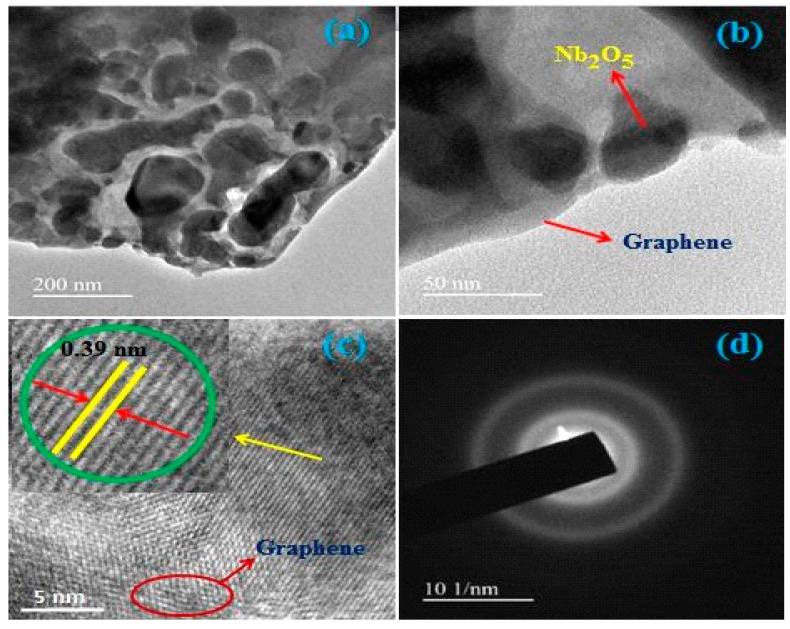
(**a**–**c**) HRTEM images of Nb_2_O_5_/G nanocomposite, and (**d**) SAED pattern.

HR-TEM images of Nb_2_O_5_ nanoparticles homogeneously anchored on the G surface are shown in Figure 3a,b. The decoration of Nb_2_O_5_ nanoparticles onto a significantly shaped crumpled sheet indicates the intercalation of Nb_2_O_5_ into the G layers controlling the re-stacking of G during the reduction process. Figure 3c shows the Nb_2_O_5_ nanoparticles with lattice spacing of 0.39 nm corresponding (001) plane of orthorhombic structure, consistent with the results of XRD analysis [22]. The SAED pattern show in Figure 3d confirms the highly crystalline nature of Nb_2_O_5_ nanoparticles. The Nb_2_O_5_ nanoparticles are well dispersed, ensuring the formation of a homogeneous composite structure. The morphology of nanocomposite provides sufficient electrochemically active sites, subsequently improving the electrochemical properties.

### 3.4. Analysis of Functional Groups 

**Figure 4:**
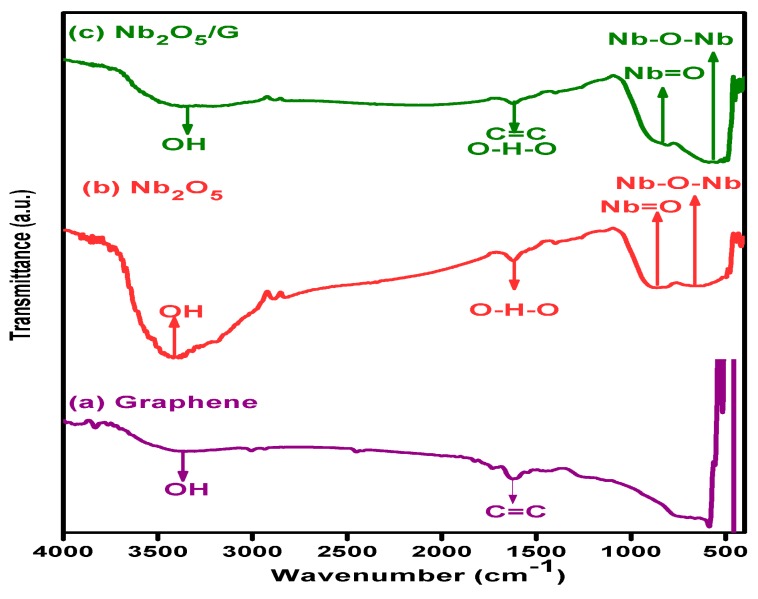
FTIR spectra of (**a**) graphene, (**b**) Nb_2_O_5_, and (**c**) Nb_2_O_5_/G nanocomposite.

Figure 4 shows the FTIR vibrational spectra of G, Nb_2_O_5,_ and Nb_2_O_5_/G nanocomposites. Figure 4a shows the spectrum for graphene with the absorption peak at 1643 cm^–1^ and broad absorption stretch at 3427 cm^–1^, which are attributed to the C=C skeletal vibration and atmospheric moisture. In Figure 4b, the absorption peaks at 3413 and 1623 cm^–1^ correspond to the stretching vibration of -OH groups due to adsorbed H_2_O. The wide band at 640 cm^–1^ is ascribed to the symmetric stretching of Nb-O-Nb, and the shoulder peak at 853 cm^−1^ corresponds to asymmetric stretching of Nb=O bands, indicating the crystalline nature of Nb_2_O_5_ [26,27]. The peaks at 3415 cm^–1^ and 1622 cm^–1^ are assigned to the O-H stretching and C=C skeletal vibration of graphene sheets in the Nb_2_O_5_/G nanocomposite (Figure 4c). The peak intensity is mostly decreased for the composite owing to the reduction of GO to G and signifying a very strong interaction between Nb_2_O_5_ nanoparticles and residual surface hydroxyl groups. 

### 3.5. Optical Properties

**Figure 5:**
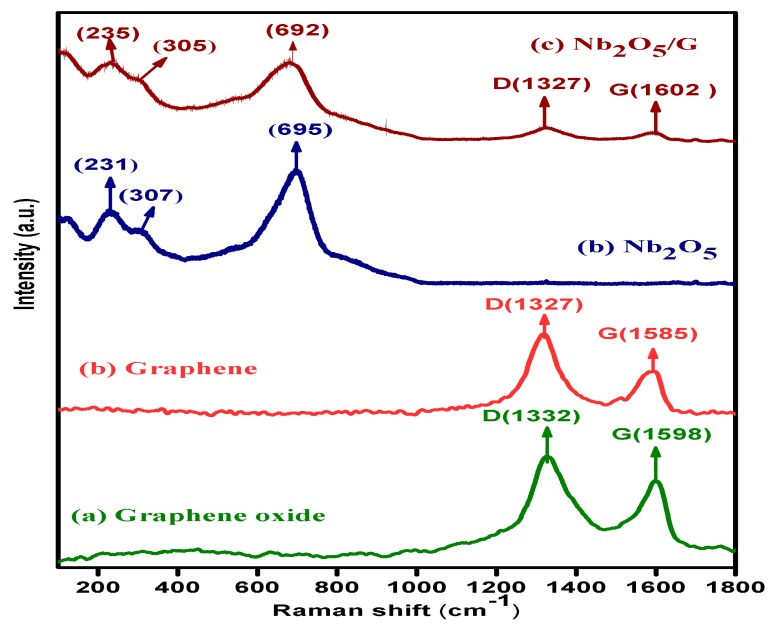
Raman spectra of (**a**) graphene oxide (**b**) graphene, (**c**) Nb_2_O_5_, (**d**) Nb_2_O_5_/G composite.

Figure 5 depicts the Raman spectra of GO, graphene, Nb_2_O_5_ and Nb_2_O_5_/G nanocomposite. Figure 5a shows the spectrum for GO with two bands centered at 1332 and 1598 cm^−1^ as prominent D and G bands respectively. Figure 5b shows the scattering spectrum of graphene with the D band (1327) related to the disorder band of sp^3^ carbon and G (1585) band corresponding to sp^2^ bonded carbon atmos. Compared with graphene oxide, a shift in the lower band was observed for the graphene, indicating the strong reduction of GO. Figure 5c depicts the spectrum of Nb_2_O_5_ with broad band at 695 cm^–1^ assigned to the symmetric and asymmetric stretching mode of Nb-O bond linkage. The peaks at 231 and 307 cm^–1^ are characteristics of Nb-O-Nb bonds, confirming the orthorhombic phase of Nb_2_O_5_ nanoparticles [28]. The Raman spectrum of Nb_2_O_5_/G composite is red shifted compared to pure graphene, providing evidence for the interactions between Nb_2_O_5_ nanoparticles and graphene sheets, as shown in Figure 5d.

### 3.6. Thermal Behavior

**Figure 6:**
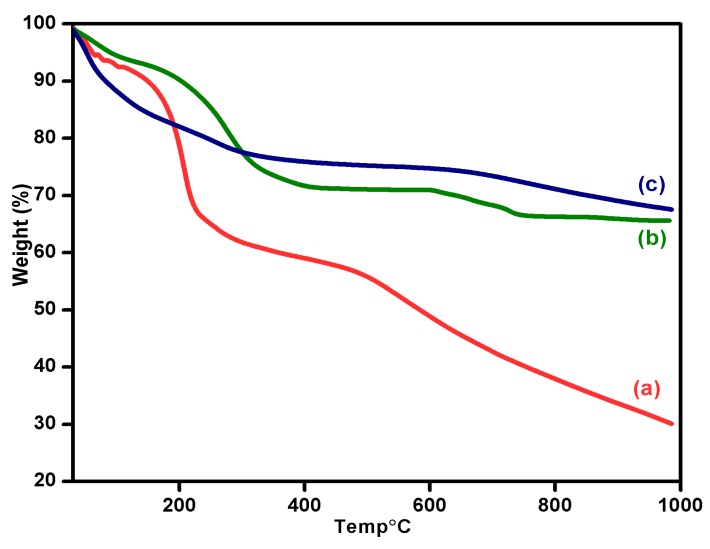
Thermogravimetric curves of (**a**) graphene oxide, (**b**) Nb_2_O_5_, and (**c**) Nb_2_O_5_/G nanocomposite.

Figure 6 shows the thermal behavior of the synthesized GO, Nb_2_O_5_ and Nb_2_O_5_/G composite investigated by TGA carried out in N_2_ atmosphere. Figure 6a shows the weight loss due to the removal of adsorbed water molecules at 100 °C and the organic matter originating from the GO in the temperature range from 150 to 250 °C. Further increasing the temperature, gradual weight loss occurred up to 1000 °C due to the liable oxygen containing functional groups with a total weight loss of 70%. Figure 6b shows the TGA curve of Nb_2_O_5_, started with a minor step from RT to 1000 °C, which indicates the release of H_2_O molecules. The next strong weight loss of 22.8% perceived from 150 to 300 °C reflects the disintegration of organic molecules. The weight loss of 2.2% at 600 °C was associated with the recrystallization due to the transformation of niobium pentoxide hydrate to a pseudohexagonal phase of Nb_2_O_5_ nanoparticles [29]. The weight loss of less than 0.5% from 800 to 1000 °C means the morphology deviation and a change in the crystal structure from pseudohexagonal to orthorhombic phase, with an overall weight loss of 34.5%. The Nb_2_O_5_/G nanocomposite possesses gradual weight loss up to 600 °C and above of 800 °C, similar behavior that of Nb_2_O_5_ was observed as shown in Figure 6c with total weight loss of 32%. The Nb_2_O_5_/G composite has the higher thermal stability and lower weight loss compared with GO and pure Nb_2_O_5_ nanoparticles.

### 3.7. Binding Energy States 

**Figure 7:**
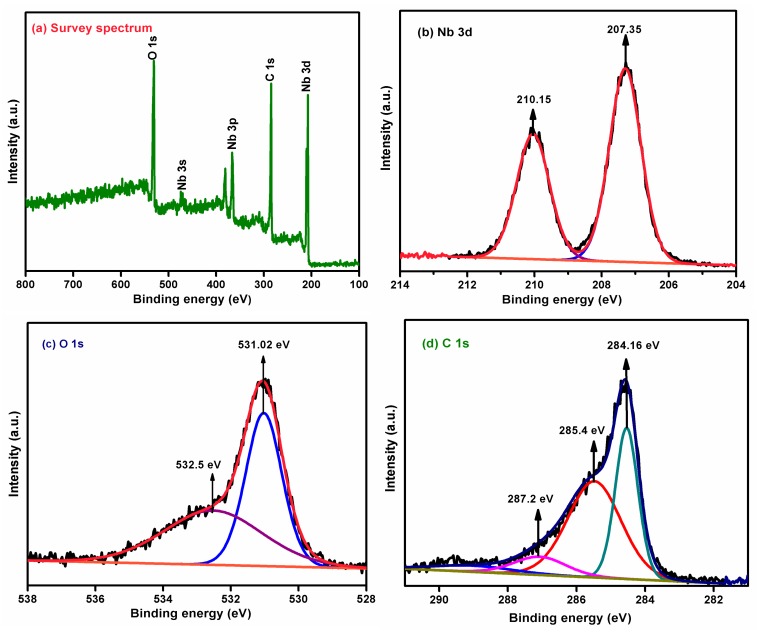
Display (XPS) the survey spectra of Nb_2_O_5_/G nanocomposite.

The XPS survey spectrum of the prepared Nb_2_O_5_/G composite with signature bands for Nb, C and O is shown in Figure 7a. This evidences the decoration of Nb_2_O_5_ nanoparticles onto the surface of G sheet. Figure 7b shows the high-resolution Nb3d spectra with binding energy of 207.35 eV (3d_5/2_) and 210.15 eV (3d_3/2_), signifying that Nb exists in the Nb^5+^ chemical state [30]. Figure 7c depicts the O1s spectrum of Nb_2_O_5_/G composite with two peaks for oxygen; first one at 531.02 eV consistent with presence of lattice oxygen in Nb_2_O_5_ and second one at 532.5 eV due to lattice oxygenated surface of the G. Figure 7d displays the high-resolution C1s spectra of Nb_2_O_5_/G with three peaks for various carbon family sp^2^ bonded carbonyls at 284.16 eV (C=C), epoxy/hydroxyl groups at 285.4 eV (C-C) and carbonyls at 287.2 eV (C=O). The chemical interaction between graphene and Nb_2_O_5_ nanoparticles is attributed to the construction bonds of Nb-O-C [31].

### 3.8. Surface Area Analysis 

**Figure 8:**
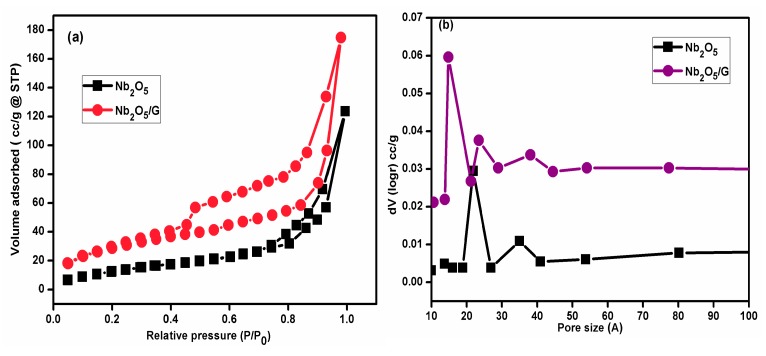
(**a**) Characteristics of N_2_ adsorption-desorption isotherm plots; and (**b**) BJH pore size distribution of Nb_2_O_5_ and Nb_2_O_5_/G nanocomposite.

The surface area analysis results (Figure 8a) reveal that both the samples exhibit a Type IV isotherm with a hysteresis loop of H4 in the P/P0 range of 0.4 to 1.0, suggesting the characteristics of a mesoporous structure. The specific surface area of the Nb_2_O_5_/G nanocomposite is higher (94 m^2^g^−1^) than that of pure Nb_2_O_5_ nanoparticles (32 m^2^g^−1^). The Specific Surface Area (SSA) of graphene was estimated to be 145.05 m^2^g^−1^ [32]. This is attributed to the porosity of G and Nb_2_O_5_ nanoparticles deposited on G sheets, which prevents the G sheets from aggregating and restacking after the removal of solvents. This leads to a porous structure of Nb_2_O_5_/G nanocomposite, and hence to a higher surface area [33]. The porous structure facilities the more efficient diffusion of electrolyte ions to the active sites. Figure 8b indicates the pore size distribution of 3.5 nm and 4.5 nm for the composite and pure Nb_2_O_5_, respectively. This mesoporous structure and high surface area of the composite play an important role in providing shorter diffusion paths, rapid electrolyte transport and additional active sites for electrochemical reaction on the electrode surface to enhance the electrochemical performance [34].

### 3.9. Electrochemical Evaluation 

#### 3.9.1. Cyclic Voltammogram 

**Scheme 1:**
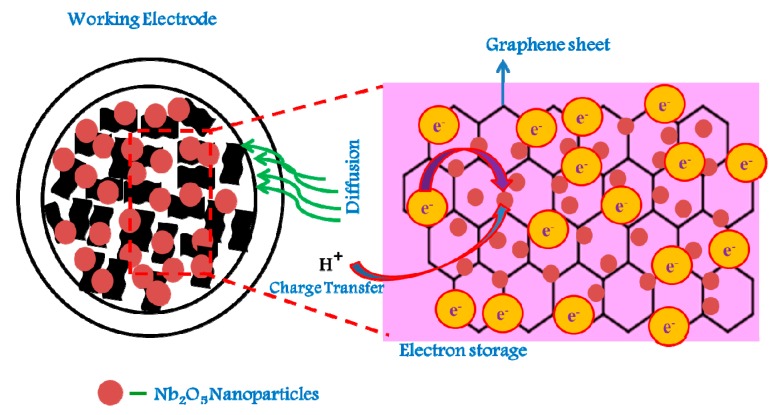
Schematic of the charge-storage mechanism of the Nb_2_O_5_/G composite electrode.

**Figure 9:**
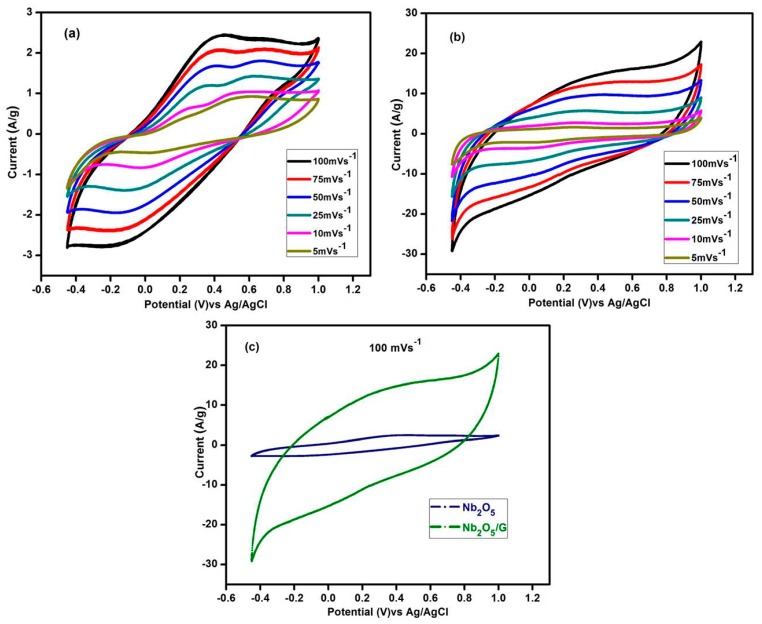
Cyclic voltamograms of (**a**) Nb_2_O_5_, (**b**) Nb_2_O_5_/G nanocomposite at different scan rates, and (**c**) comparison of CV curves of Nb_2_O_5_, and Nb_2_O_5_/G at a scan rate of 100 mVs^−1^.

Figure 9a displays the typical cyclic voltammogram (CV) curves of the Nb_2_O_5_ electrode material for the potential range from −0.45 to 1.0 V at various scan rates in 1 M H_2_SO_4_ aqueous electrolyte. The CV curves of Nb_2_O_5_ exhibit oxidation reduction peaks for the sweep rate from 5 to 100 mVs^−1^, which are evidently the characteristics of Faradic behavior. With the increasing scan rate, the redox peaks become stable, indicating a strong kinetic reversibility. At higher scan rates the increase in the anodic peak current density with a decrease in the cathodic peak indicates the low resistance of the electrode. The CV curves shown in Figure 9b exhibit a quasi-rectangular shape without oxidation and reduction peaks, suggesting the higher stability of Nb_2_O_5_/G composite electrodes for a wider potential range. Moreover, the quasi-rectangular shape of CV curves reveals exceptional reversibility and faster surface reaction. This confirms the ideal capacitive behavior with excellent electrochemical property and electrical conductivity. The CV curves recorded at low scan rates are due to the strong reversible process. These results confirm that the Nb_2_O_5_/G composite electrode has excellent capacitance behavior and low contact resistance, provided by high surface area. Figure 9c shows the comparison of CV curves of Nb_2_O_5_ and Nb_2_O_5_/G composites electrodes at a scan rate of 100 mVs^−1^. The composite electrode showed a higher integral area than the pure Nb_2_O_5_ electrode, confirming the higher specific capacitance. In the composite electrodes the ionic charge accumulates at the electrode/electrolyte interface due to high surface area and the porosity of the G. The higher capacitance suggests that Nb_2_O_5_ nanoparticles might be intercalated at the pores to support the EDLC formation. The charge storage mechanism of Nb_2_O_5_/G composites is shown in Scheme 1.

#### 3.9.2. Chronopotentiometry Measurements

**Figure 10:**
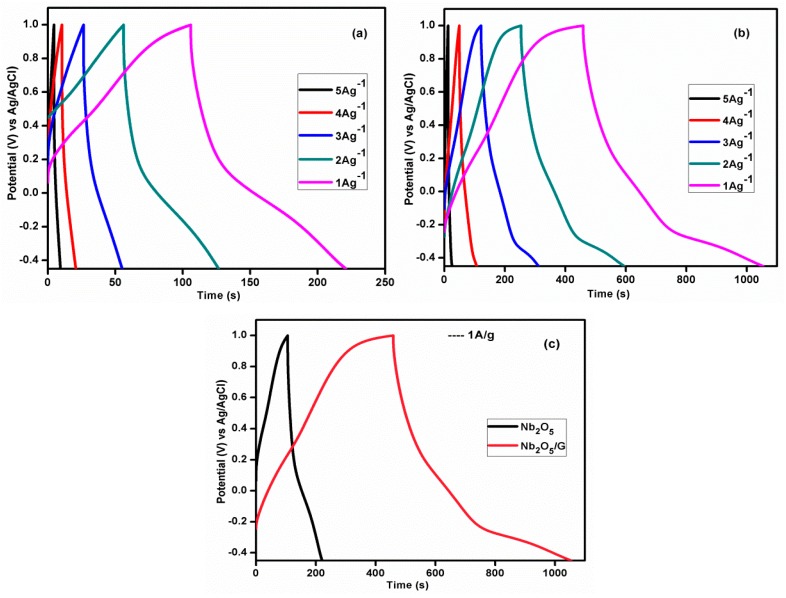
Charge-discharge profile of (**a**) Nb_2_O_5_ and (**b**) Nb_2_O_5_/G nanocomposites. (**c**) Comparison charge-discharge profile at a current density of 1 Ag^−1^.

The charge-discharge performance was analyzed in the potential range from 0.45 to 1.0 V vs. Ag/AgCl, in 1 M H_2_SO_4_ aqueous electrolyte. Figure 10a shows the charge-discharge curves of Nb_2_O_5_, and displays nonlinear and symmetrical shapes for the corresponding discharge period with a slight plateau without internal resistance (IR) drop. The charge and discharge platforms correspond to the oxidation and reduction of Nb_2_O_5_ nanoparticles. While increasing the current density, the charge-discharge process decreases gradually due to the adsorption/desorption of H^+^ ions during the charge-discharge process. The CP curves of the Nb_2_O_5_/G electrode without linear and symmetrical shapes confirm the characteristics of non-Faradic behavior at different current densities are shown in Figure 10b. The perfect symmetry of the CP curves indicates represents the excellent reversibility in non-Faradic reactions. The discharge portion displays a longer discharge time at low current density, indicating higher capacitance. The decrease in current density and increased discharge time are due to the higher surface area and conductivity of graphene. The CP curves show no obvious IR drop due to the low thermal resistance, well-formed electrode/electrolyte interface and high reversibility and good capacitive nature of the electrode materials. 

Figure 10c displays the comparison CP curves of Nb_2_O_5_ and Nb_2_O_5_/G nanocomposite at a current density of 1 Ag^−1^. The composite exhibits higher discharge time than pure Nb_2_O_5_ electrode. The Nb_2_O_5_ nanoparticles possess poor electrical conductivity and fast degradation as compared to the Nb_2_O_5_/G composite electrode material. The combination of Nb_2_O_5_ nanoparticles with G increases the conductivity thereby effectively creating conducting paths for the electrons to achieve ideal capacitive behavior. Thus, increasing the overall discharge time of composite electrodes is beneficial to improving the specific capacitance by shortening the diffusion and moving length of the electrolyte ions. The G ensures the strong conductive network for the ions transport due to the higher interfacial contact area between Nb_2_O_5_ and G. The specific capacitance (C_s_) values of pure Nb_2_O_5_ and Nb_2_O_5_/G nanocomposites were obtained from CP curves using the formula,

C_s_ = I × Δt/m × ΔV (1)

where, I—discharge current, ΔV—potential window, t—discharge time, m—mass of the active material in the electrode. The calculated specific capacitance values were 633, 271, 445, 314.8, 238.1, 168.9 and 221, 168.5, 82.1, 34.5, 19 Fg^−1^ for Nb_2_O_5_/G nanocomposite and pure Nb_2_O_5_ electrodes, respectively, at different current density values. The capacitance of pure graphene was measured to be 290 Fg^−1^ [35]. The specific capacitance values of the composite electrode have been compared with previously reported results as shown in Table 1.

**Figure 11:**
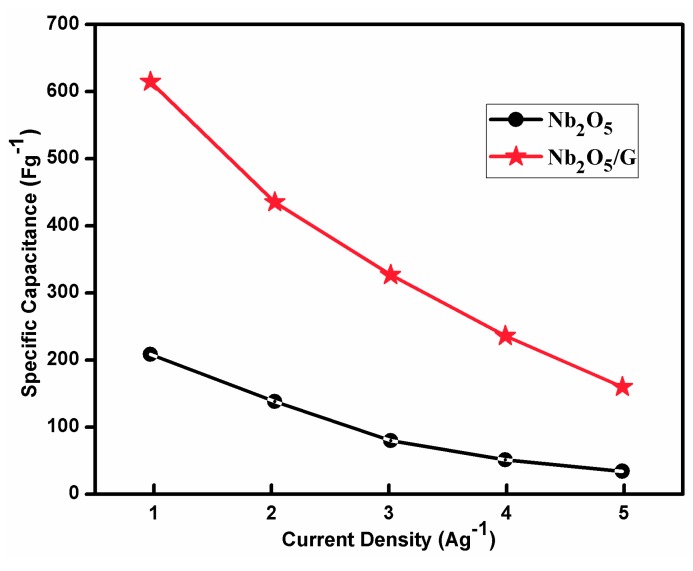
Specific capacitance of pure and composite electrode material at different current density values.

The calculated specific capacitance (Cs) values of Nb_2_O_5_ and Nb_2_O_5_/G nanocomposite electrode at different current densities are shown in Figure 11. The response of both the electrodes decreased with increasing current density from 1 to 5 Ag^−1^. The specific capacitance of the composite electrode was higher than pure Nb_2_O_5_ electrode. The enhanced capacitive performance of the composite is due to the synergistic effect of higher conductivity of G and mesopores structure of Nb_2_O_5_ nanoparticles. Additionally, well-dispersed Nb_2_O_5_ nanoparticles can avoid the restacking of G sheets to ensure higher surface area for the storage [37]. Meanwhile, Nb_2_O_5_/G nanocomposite could provide higher contact area, improving the electrochemical performance [38].

**Figure 12:**
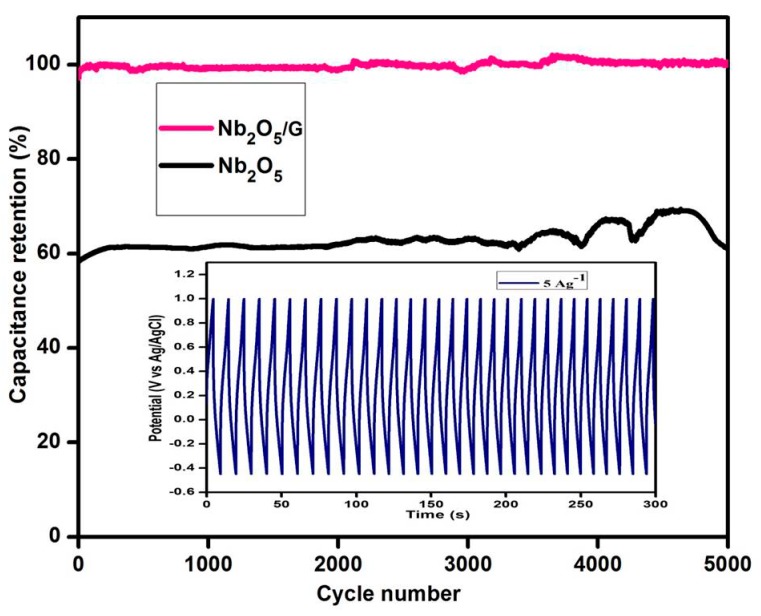
Capacitance retention of Nb_2_O_5_/G composite electrode upto 5000 cycles at a current density of 5 Ag^−1^ with an inset for a few cycles.

Figure 12 depicts the capacitance retention of the Nb_2_O_5_/G nanocomposite electrode tested up to 5000 cycles. The composite electrodes maintain a stability of 99.3% after 5000 cycles without decay, indicating the excellent electrochemical stability of electrode, which is higher than pure Nb_2_O_5_ electrode material. This is attributed to the relatively easy path for electrode/electrolyte interface reactions. Moreover, the 2D network of the G sheet with low resistance allows rapid and effective electron transport providing higher stability. The Coulombic efficiency (η) was calculated from the CP curves using the following equation,

η = (t_d_/t_c_) × 100 (2)

where t_d_ and t_c_ are the discharge time and charge time. The Nb_2_O_5_/G nanocomposite electrode retains the Coulombic efficiency of 99.5%, as derived from its symmetrical charge-discharge curves. 

#### 3.9.3. Electrochemical Impedance Analysis

**Figure 13:**
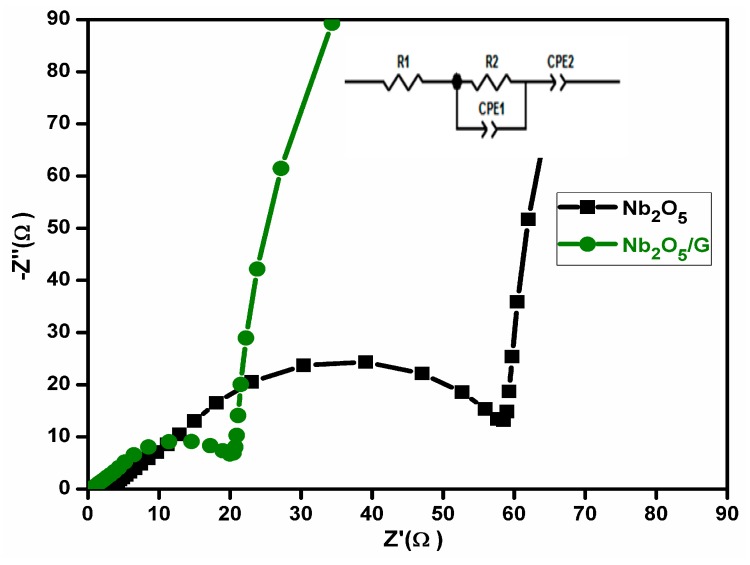
The complex impedance (Nyquist plots) of prepared Nb_2_O_5_ and Nb_2_O_5_/G composite electrodes. Inset shows the corresponding equivalent circuit.

Typical electrochemical impedance spectroscopy (EIS) plots of Nb_2_O_5_ and Nb_2_O_5_/G nanocomposite electrodes in the frequency range of 0.01 Hz to 105 Hz are presented in Figure 13. The semicircle in the high-frequency region represents the solution resistance (R_s_). The slope in the low-frequency range is attributed to Warburg resistance, which results from the frequency dependence of ions diffusion. The solution resistance (R_s_) of composite and pure Nb_2_O_5_ is measured to be 0.61 and 2.2 Ω respectively. The equivalent circuit best fits the experimental data provided in the inset of Figure 13, where R_S_—solution resistance, R_ct_—charge transfer resistance, and CPE—constant phase element values are given in Table 2. The estimated solution resistance (R_s_) value for the composite (0.61 Ω) is much lower than pure Nb_2_O_5_ (2.2 Ω), revealing the higher accessibility of active sites in composite for electrolyte ions. The composite electrodes display lower R_s_ value than the Nb_2_O_5_ due to the intrinsic electrical conductivity. The lower R_s_ value of the composite electrodes suggests that the introduction of G significantly enhances the electrical conductivity of the composite [39] due to strong interface between Nb_2_O_5_ and the G framework, which enhances the electrochemical activity. 

## 4. Conclusions

Nanocomposites of Nb_2_O_5_/G were synthesized via a facile in situ microwave irradiation method. The XRD results confirmed the reduction of graphene oxide into graphene and orthorhombic structure of Nb_2_O_5_ in the composite. The aromatic ring of C=C bands from oxygen functionalities of G appeared in the composite as confirmed from FTIR studies. The HR-TEM analysis reveals the decoration of Nb_2_O_5_ nanoparticles on G sheets. Higher specific capacitance of 633 Fg^−1^ was measured for Nb_2_O_5_/G nanocomposites much higher than pure Nb_2_O_5_ nanoparticles (221 Fg^−1^) and graphene (290 Fg^−1^) at 1 Ag^−1^. The cyclic stability of 99.3% was achieved at 5000 cycles, which confirmed that the composite electrode can be a better choice for energy storage applications for supercapacitor. 

## Figures and Tables

**Table 1:** The specific capacitance and retention of Nb_2_O_5_/G nanocomposite electrodes synthesized by different methods.

Electro-Active Material	Synthesis Method	Specific Capacitance (F/g)	Number of Cycles	Retention	Ref.
Nb_2_O_5_/graphene	Hydrothermal	34	50	~80	[36]
Nb_2_O_5_/graphene	Hydrothermal	58	50	91%	[21]
T-Nb_2_O_5_/graphene	Facile-hydrothermal	80	~3000	~100%	[22]
Graphene/Nb_2_O_5_	In situ hydrothermal method	321	500	91%	[23]
Nb_2_O_5_/graphene	In situ microwave method	633	5000	100%	Present work

**Table 2:** Calculated values of R_S_, R_ct_, CPE_1_ and CPE_2_ through fitting of the experimental impedance spectra based on the equivalent circuit in Figure 13.

Sample	R_S_ (Ω)	R_ct_ (Ω)	CPE_1-T_ (F)	CPE_1-p_ (F)	CPE_2-T_ (F)	CPE_1-p_ (F)
Nb_2_O_5_/G	0.61	20.05	0.0009	1.05	0.30	0.905
Nb_2_O_5_	2.2	58.53	0.0009	1.05	0.39	0.9
